# Nathan Drake

**Published:** 1836-07

**Authors:** 


### NATHAN DRAKE, M.D.

Although a highly respectable and much respected member of the medical profession, and although distinguished in his own circle as a physician, Dr. Drake was much more known as a popular and successful cultivator of general literature. He was a very voluminous writer, as will appear from the following list of his principal productions:

Poems. *Lond.* 1793. 4to.—Literary Hours; or Sketches, Critical, Narrative, and Poetical. *Sudbury,* 1798-1800. *Lond.* 1804. 3 vols. 8vo.—Essays illustrative of the Tatler, Spectator, and Guardian. *Lond.* 1805. 3 vols. 12mo. —Essays illustrative of the Rambler, Adventurer, Idler, &c. *Buckingham,* 1809. 2 vols. 8vo.—The Gleaner; a Series of periodical Essays, selected from Papers not included in the British Essayists. 1811. 4 vols. 8vo.—Winter Nights. 2 vols. 8vo.—Shakespeare and his Times. 2 vols. 4to. 1817.—Autumn Evenings. 2 vols. 8vo.—Noontide Leisure. 2 vols. 8vo.—Mornings in Spring, 2 vols. 8vo. 1828.—Memorial of Shakespeare. 8vo.

We are not aware of Dr. Drake having published any distinct medical work. In the year 1799, he communicated to Dr. Beddoes some Observations on the use of Digitalis in Consumption, which were published in his work entitled "Contributions to Physical and Medical Knowledge;" and he contributed two papers on the same subject to the second volume of the London Medical and Physical Journal, for the same year. He also published a singular case of Diseased Spleen, in the Edinburgh Med. and Surg. Journal for 1806, vol. ii.

Dr. Drake graduated at Edinburgh in the year 1789, his thesis being *De Somno.* He spent the greater portion of his life at Hadleigh, in Suffolk, where he died on the 7th June last, in the seventy-first year of his age. As a medical practitioner, Dr. Drake was deservedly esteemed by his professional brethren for his courtesy and skill; while to those whom he attended, he was yet more endeared by the urbanity of his manners and the unaffected kindness of his heart.

